# Rosiglitazone as a Modulator of TLR4 and TLR3 Signaling Pathways in Rat Primary Neurons and Astrocytes

**DOI:** 10.3390/ijms19010113

**Published:** 2018-01-02

**Authors:** Dmitry V. Chistyakov, Nadezda V. Azbukina, Alexandr V. Lopachev, Ksenia N. Kulichenkova, Alina A. Astakhova, Marina G. Sergeeva

**Affiliations:** 1 Belozersky Institute of Physico-Chemical Biology, Moscow State University, Moscow 119992, Russia; alina.an.astakhova@gmail.com (A.A.A.); mg.sergeeva@gmail.com (M.G.S.); 2 Laboratory of electrophysiology, Pirogov Russian National Research Medical University, Moscow 117997, Russia; 3 Faculty of Bioengineering and Bioinformatics, Moscow Lomonosov State University, Moscow 119234 Russia; ridernadya@gmail.com; 4 Research Center of Neurology, Moscow 125367, Russia; lopsasha@yandex.ru (A.V.L.); koulitchenkova@gmail.com (K.N.K.)

**Keywords:** toll-like receptor (TLR), poly I:C (PIC), lipopolysaccharide (LPS), interleukin-10 (IL-10), tumor necrosis factor alpha (TNFα), mitogen-activated kinase (MAPK), rosiglitazone (RG), thiazolidinedione (TZD)

## Abstract

An antidiabetic drug of the thiazolidinedione class, rosiglitazone (RG) demonstrates anti-inflammatory properties in various brain pathologies. The mechanism of RG action in brain cells is not fully known. To unravel mechanisms of RG modulation of toll-like receptor (TLR) signaling pathways, we compare primary rat neuron and astrocyte cultures stimulated with the TLR4 agonist lipopolysaccharide (LPS) and the TLR3 agonist poly I:C (PIC). Both TLR agonists induced tumor necrosis factor (TNFα) release in astrocytes, but not in neurons. Neurons and astrocytes released interleukin-10 (IL-10) and prostaglandin E2 (PGE_2_) in response to LPS and PIC. RG decreased TLR-stimulated TNFα release in astrocytes as well as potentiated IL-10 and PGE_2_ release in both astrocytes and neurons. RG induced phosphorylation of p38 and JNK MAPK (mitogen-activated protein kinase) in neurons. The results reveal new role of RG as a modulator of resolution of neuroinflammation.

## 1. Introduction

Neuroinflammation is a form of innate immune response initiated by altered homeostasis within brain tissues. Neuroinflammation accompanies all known neurological pathologies, including neurodegenerative diseases; post-ischemic neurodegeneration; and traumatic, metabolic, toxic and neoplastic disturbances [1,2,3]. Although neuroinflammation has protective functions, its intrinsic cytotoxicity is recognized among major factors exacerbating brain pathologies of the brain. Thus, detailed examination of neuroinflammation on cellular and molecular levels is crucial for new therapeutic targets discovery and development of effective treatment strategies.

Toll-like receptor mediated signaling cascades play crucial role in peripheral inflammatory responses. Toll-like receptors (TLRs) can be activated both by various exogenous ligands (pathogen-associated molecular pattern or PAMP) and by an array of endogenous molecules generated and released during tissue damage (damage associated molecular pattern or DAMP) [4]. The mammalian TLR family is subdivided into two main groups: (1) cell surface TLRs (TLR1, -2, -4, -5, -6, and -10) that recognize PAMPs that are mainly constituent of the bacterial cell wall or are expressed on the bacterial cell surface, such as lipopolysaccharide (LPS) (TLR4 agonist); and (2) intracellular TLRs (TLR3, -7, -8, and -9) that recognize microbial nucleic acid, such as double-strand RNAs (TLR3). Activation of the TLR signaling pathways induces mitogen-activating protein kinases (MAPKs) and transcription factors, such as NF-κB, AP-1, IRF-3, etc., and ultimately results in release of various cytokines and signaling lipids [5,6,7]. Currently, TLRs themselves and multiple components of TLR pathways are considered as the most promising targets for control of inflammation in many disorders.

TLRs are deeply involved in neuroinflammation as well. Notably, not just microglia, but all cells of the central nervous system (CNS) contribute to neuroinflammation through activation of TLR-mediated cascades [2,8,9,10]. Understanding TLR pathway regulation in nervous cells of non-immune origin is one of the crucial questions of neuroinflammatory control.

Rosiglitazone (RG) is a drug of thiazolidinediones (TZDs) class that has demonstrated positive effects in various investigations related to brain pathologies, including in vivo studies of Alzheimer’s disease [11], models of Parkinson’s disease [12] and Huntington’s disease [13]. RG was also effective in preventing cell death after cerebral ischemia in animal models [14]. These effects were attributed to anti-inflammatory activity of RG on the CNS-resident cells level [15]. Although effects of RG on neuron response to pro-inflammatory challenges have not been investigated thus far, anti-inflammatory characteristics of TZDs were demonstrated for astroglial and microglial cells in several of studies. RG and other TZDs were able to inhibit inflammatory activation of cultured astrocytes and microglia by diminishing LPS-induced pro-inflammatory cytokines (interleukin 6, tumor necrosis factor α (TNFα)), and other markers of inflammatory cellular responses [16,17,18,19]. These effects of TZDs were mediated by activation of peroxisome proliferator-activated receptor-γ (PPAR-γ) [16] or kinase phosphorylation [20]. Recently, we have indicated another mechanism of RG mediated effects. We have shown that RG treatment increased LPS-induced interleukin 10 (IL-10) mRNA expression via an increase of IL-10 mRNA half-life [21]. Taken together, these data point to possible role of RG in the resolution phase of a neuroinflammatory response and raises a question of RG effect on TLR-induced pro-inflammatory responses of neurons in primary cultures. 

The aim of our investigation was to assess the contribution of neurons to anti-inflammatory and resolution effects of rosiglitazone. We choose LPS, a TLR4 agonist, as a representative of cell surface receptors, and Poly I:C (PIC), a TLR3 agonist, as a representative of endosomal receptors. Their effects on release of the pro-inflammatory cytokine TNFα; the cytokine of the resolution phase IL-10; and prostaglandin E2 (PGE_2_) in neurons were analyzed. Modulation of kinase activity (p38 MAPK and JNK phosphorylation) was estimated as a possible intracellular mechanism of RG action. The data of TLR-stimulated neurons were compared with data of the same astrocyte stimulation. The results reveal a new role of RG as a modulator of neuroinflammation resolution.

## 2. Results

**Figure 1:**
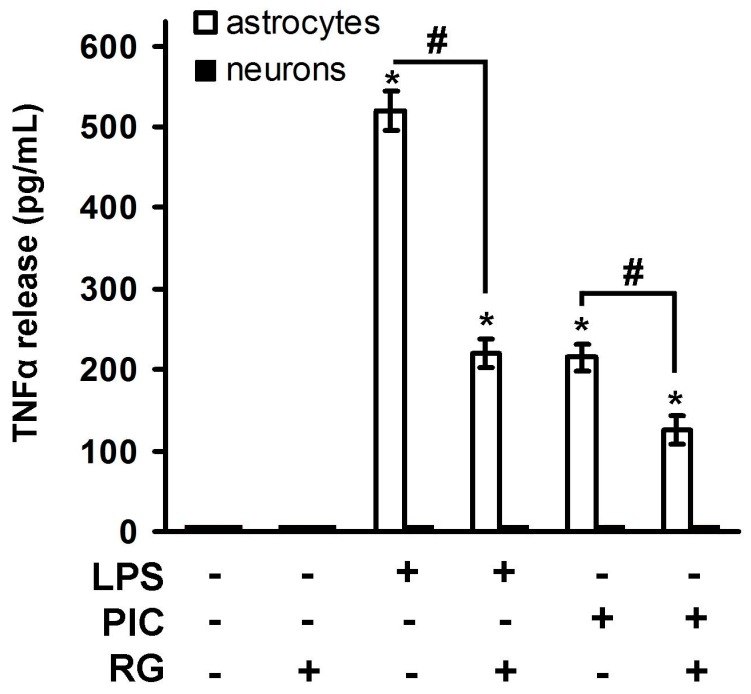
Toll-like receptor (TLR) agonists induce tumor necrosis factor α (TNFα) release in astrocytes and neurons. Astrocytes (white) and neurons (black) were pretreated for 0.5 h with rosiglitazone (RG, 10 μM) and subsequently kept for 4 h with lipopolysaccharide (LPS, 100 ng/mL) or Poly:IC (PIC, 10 μg/mL). TNFα concentrations were measured by enzyme-linked immunosorbent assay (ELISA) in supernatant samples of three cell cultures. The detection limit of the ELISA was 15 pg/mL. Values represent mean SEM from three independent experiments performed in triplicate. * *p* < 0.05 compared with the unstimulated cells, # *p* < 0.05 compared indicated bars.

TLR4 and TLR3 mediated release of TNFα by astrocytes has already been clearly shown [22,23,24]. Some data obtained from nervous tissue pointed that neurons could also express TNFα (both on mRNA and protein levels) [25,26], however data at the cellular level are still missing. Therefore, we estimated concentration of TNFα in extracellular samples 4 h after stimulation of neuronal and astrocytic cultures with TLR3 and TLR4 agonists (Figure 1). Both PIC and LPS induced TNFα release in astrocytes, but not in neurons. It is known that TNFα reaches its maximum concentration 2–4 h after a pro-inflammatory challenge and then is eliminated from extracellular media in a specific time depending on a cell type [27]. Thus, as we had not detected TNFα in neuronal cultures within the optimal time frame it is unlikely that neurons release TNFα at the later time points. To this end, longer periods of TLR stimulation were not analyzed. Treatment with RG downregulated TLR-induced TNFα release in astrocytes stimulated with both the TLR4 and TLR3 agonists (Figure 1). We detected no TNFα release in naive cells, whereas addition of LPS or PIC to astrocytes increased the TNFα levels to 500 and 220 pg/mL, respectively (Figure 1). RG significantly decreased the TNFα synthesis shifting the levels to 212 and 120 pg/mL, respectively (Figure 1). Taken together, these data demonstrate that the LPS and PIC treatment induced release of TNFα by astrocytes, but not by neurons.

### 2.1. Rosiglitazone Regulation of IL-10 Expression upon Activation of TLR3 and TLR4 Receptor in Astrocytes and Neurons

**Figure 2:**
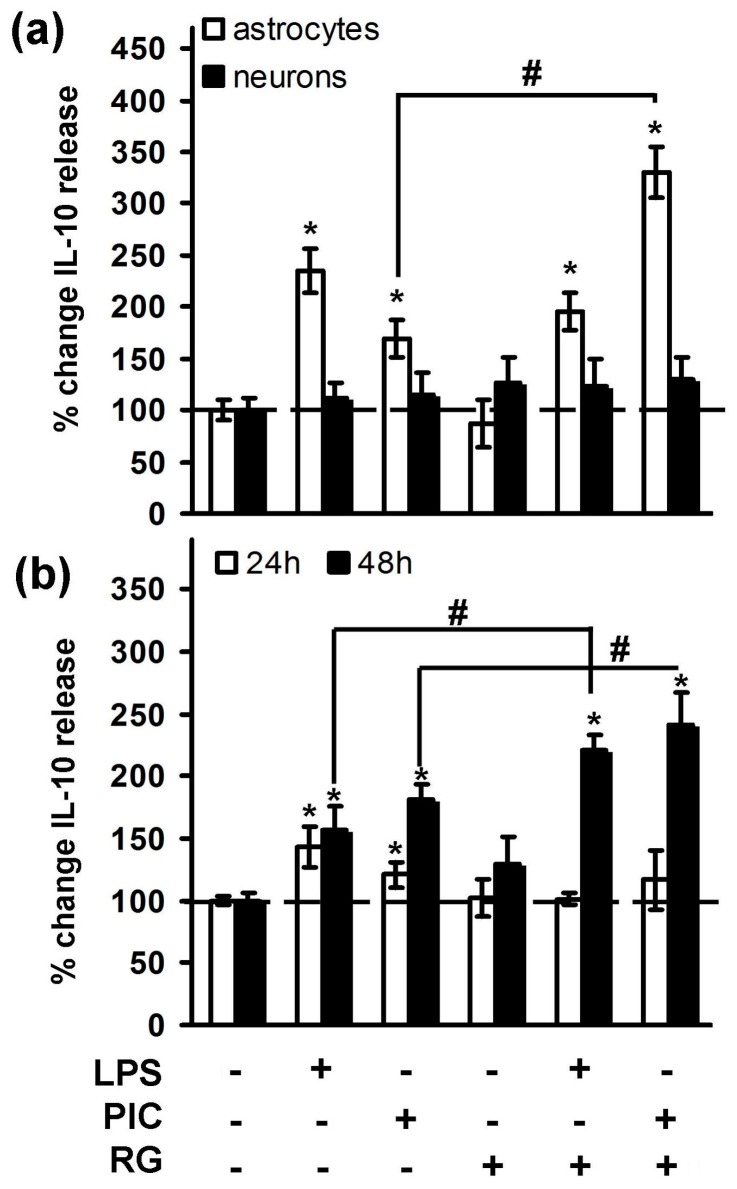
Rosiglitazone modulates IL-10 release at the protein level in astrocytes and neurons. (**a**) Astrocytes and neurons were pretreated for 0.5 h with rosiglitazone (RG, 10 μM) and subsequently kept for 4 h with lipopolysaccharide (LPS, 100 ng/mL) or Poly:IC (PIC, 10 μg/mL). (**b**) Neurons were pretreated for 0.5 h with rosiglitazone (RG, 10 μM) and subsequently kept for 24 h or 48 h with lipopolysaccharide (LPS, 100 ng/mL) or Poly:IC (PIC, 10 μg/mL). IL-10 concentrations were measured by ELISA in two supernatant samples of three cell cultures. IL-10 data are calculated as nanograms per million cells and expressed as a percentage of control (100%). Values represent mean ± standard error of the mean (SEM) from three independent experiments performed in triplicate. * *p* < 0.05 compared with the unstimulated cells, # *p* < 0.05 compared indicated bars.

It was shown, that LPS-induced IL-10 expression on both mRNA [21] and protein levels [28,29] in astrocytes to promotes neuronal survival after spinal cord injury [30]. There has been no data concerning PIC stimulation of astrocytes and effects of both TLR agonists in neurons. Therefore, we estimated concentration of IL-10 in extracellular media after 4 h of cell stimulation with TLR3 and TLR4 agonists and compared neuron and astrocyte cell cultures (Figure 2a). Both PIC and LPS induced IL-10 release in astrocytes (up to two-fold), but not in neurons. Noteworthy, RG significantly potentiated PIC-induced IL-10 release (Figure 2a). TLR-stimulated IL-10 release had prolonged pattern, therefore we measured the levels of the cytokine at the time points of 24 and 48 h after LPS and PIC stimulation of neurons (Figure 2b). Indeed, both TLR agonists induced IL-10 release at 48 h and RG potentiated action of PIC and LPS up to 2.5-fold. Thus, one of the protective mechanisms of rosiglitazone action in activated brain cells relates to upregulation of IL-10, an important cytokine of the resolution phase of inflammation.

### 2.2. The Effect of Rosiglitazone on PGE_2_ Release upon Activation of TLR3 and TLR4 in Astrocytes and Neurons

**Figure 3:**
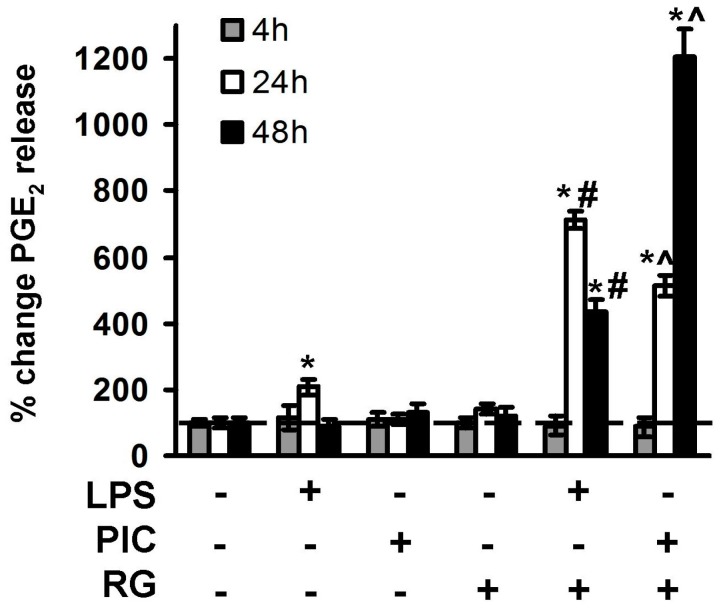
Rosiglitazone modulate PGE_2_ release in cortex neurons. Neurons were pretreated for 0.5 h with rosiglitazone (RG, 10 μM) and subsequently kept for 4 h, 24 h or 48 h with lipopolysaccharide (LPS, 100 ng/mL) or Poly:IC (PIC, 10 μg/mL). PGE_2_ concentrations were measured by ELISA in two supernatant samples of three cell cultures. PGE_2_ data are calculated as nanograms per million cells and expressed as a percentage of control (100%). Values represent mean ± SEM from three independent experiments performed in triplicate. * *p* < 0.05 compared with the unstimulated cells, # *p* < 0.05 compared indicated bars, ^ *p* < 0.05 compared with the 24 h stimulated cells.

Induction of prostaglandin synthesis by TLR agonists is typical for macrophages and other types of brain cells [31]. Initially, release of prostaglandin E2 (PGE_2_) was considered as an inflammatory marker, but, recently, it has become obvious that TLRs are responsible for synthesis of both pro-inflammatory signaling lipids and lipids of resolution [32]. It is known that RG modulates PGE_2_ release in astrocytes [17,21,24], but involvement of RG in IL-10 regulation in neurons remains unexplored. Therefore, we evaluated influence of RG on LPS- and PIC-stimulated release of PGE_2_ 4, 24 and 48 h after challenge (Figure 3). Significant concentrations of PGE_2_ in extracellular media were detected 4 and 24 h after LPS stimulation, but not after treatment with PIC. When added alone, PIC did not induce PGE_2_ synthesis at the tested time points (Figure 3). Addition of RG 0.5 h prior to TLR stimulation downregulated LPS-induced PGE_2_ release at the time point of 4 h, but significantly enhanced PGE_2_ release at the time points of 24 and 48 h for both LPS and PIC stimulations (Figure 3). The concentrations of PGE_2_ (pg/mL) were 204 ± 37 (RG + LPS) and 567 ± 39 (RG + LPS) at 48 h.

### 2.3. The Effect of Rosiglitazone on the Phosphorylation of JNK and p38 MAPK in Neurons

**Figure 4:**
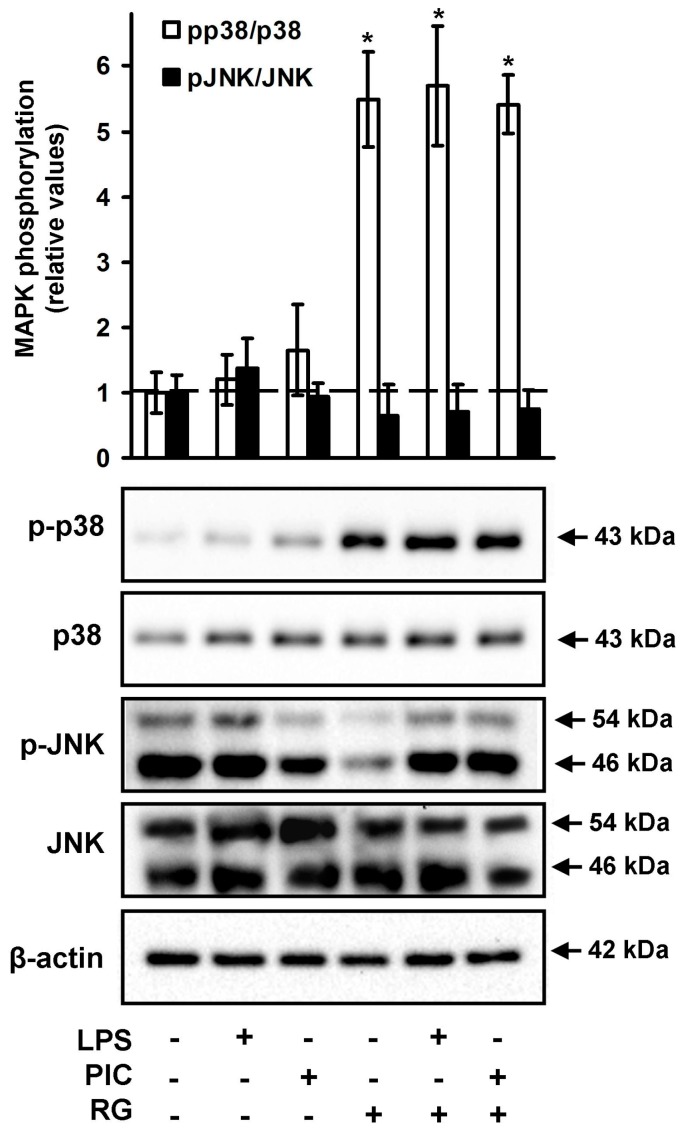
Rosiglitazone increases p-p38 but not p-JNK levels in cortex neurons. Neurons were pretreated for 0.5 h with rosiglitazone (RG, 10 μM) and subsequently kept for 4 h with lipopolysaccharide (LPS, 100 ng/mL) or Poly:IC (PIC, 10 μg/mL), after that total cell lysates were harvested. Activation of JNK and p38 was determined by Western blotting using phospho-specific antibodies. Equal protein loading was confirmed using b-actin antibody. The blot is representative of three independent experiments. * *p* < 0.05 compared with the unstimulated cells.

Activation of TLR signaling pathways activates members of the major mitogen-activated protein kinase (MAPK) subfamilies, among them p38 and Jun N-terminal kinase (JNK) [6]. Previously rosiglitazone was demonstrated to modulate TLR/MAPK signaling pathways in other cell types [20]. Therefore, we tested p38 MAPK and JNK expression and phosphorylation during LPS- and PIC-induced responses of neurons (Figure 4). Rosiglitazone significantly induced levels of p38 phosphorylation both being added alone or with TLR4 or TLR3 agonists (Figure 4). The levels of both phospho-JNK and total JNK (Figure 4) migrating at two molecular weights of p54 and p46 are demonstrated from the same samples. We observed that pre-incubation with RG of TLR-stimulated cells did not significantly affect the levels of phosphorylation of both p46 and p54 subunits for JNK. These data suggested a link between rosiglitazone and p38 MAPK signaling pathways in neurons.

## 3. Discussion

The novelty of the present research relates to the study of TLR3- and TLR4-mediated pathways in cortex neurons. Our data indicate that neurons do not produce TNFα in a response to exogenous stimuli (TLR3 agonist, PIC; TLR4 agonist, LPS), but release IL-10 after prolonged (more than 24 h) incubation with these TLR agonists. 

It has become clear recently that stimulation of inflammatory receptors, for instance TLRs, does not simply result in release of the pro-inflammatory mediators (such as TNFα and PGE_2_), but initiates protective processes as well. To emphasize the importance of these protective mechanisms, the term “resolution” has been introduced. Currently, resolution names a period between the peak of an inflammatory cell influx and elimination of these cells from a damaged tissue site followed by functional homeostasis restoration [7]. IL-10 is a key anti-inflammatory cytokine that inhibits induction of pro-inflammatory cytokines, including TNFα and IL-6 [33], and is considered among the most important cytokines of the inflammation resolution phase [34]. Our finding that IL-10 is present in neuron culture media 24 and 48 h after stimulation, during the time frame that is attributed to the resolution phase (24 and 48 h after stimulation) comes into agreement with the idea of pro-resolution functions of IL-10.

The majority of investigations with rosiglitazone in the brain were focused on its neuroprotective effects in various models. Repeated treatments with rosiglitazone demonstrated neuroprotective effects in the brain and retina [12]. These results were obtained using tissue-dissolving histological analysis. Another research group [35] used a rat model of global cerebral ischemia with rapid local RG injections into the damaged sites and found that RG attenuated inflammation and neuronal loss. Molecular mechanisms of RG action in neuronal cells remained undiscovered. In the present work, we have demonstrated that treatment with RG influenced TLR-mediated release of PGE_2_ and IL-10 in neurons. TNFα expression on both mRNA and protein levels has been previously detected in cerebral cortex of rats. Expression of this peptide appears to facilitate infiltration of inflammatory cells into sites of injury that can further exacerbate tissue damage during cerebral ischemia and contribute to increased sensitivity and risk of focal stroke [26]. Our results indicate that other CNS resident cells rather than neurons could be responsible for TNFα production rather than neurons. We were able to detect significant expression of TNFα in astroglial cultures treated with TLR3 and TLR4 agonists and these data came into agreement with previously reported results of the other studies [22,23,24]. According to our results, pre-incubation with RG leads to downregulation of TNFα secretion into media in LPS- or PIC-activated astroglial cultures. This effect can be interpreted as an anti-inflammatory and neuroprotective one.

Our data indicated significant upregulation of PGE_2_ production in LPS and PIC challenged neurons pretreated with RG. Induction of COX-2 enzymatic activity is generally considered among detrimental factors within the course of neuroinflammation. This conclusion is based upon observations that pharmacological inhibition of COX-2 activity leads to reduction of PGE_2_ levels and provides neuroprotection [36,37]. However, several studies come into contradiction with this statement. Indeed, PGE_2_ was demonstrated to elicit a neuroprotective effect in dispersed pure neurons [38]. In another study, PGE_2_ protected neurons from LPS-induced apoptosis via modulation of ROS production [39]. Interestingly, concentrations used in this investigation were significantly higher (1 μg/mL) than those detected in our models (RG together with LPS or PIC induced PGE_2_ up to 567 pg/mL). Induction of PGE_2_ by rosiglitazone in LPS-stimulated astrocytes was previously attributed to a possible positive role of rosiglitazone as anti-inflammatory substance [17]. Finally, PGE_2_, as well as other primary prostaglandins, is known to turn into cyclopentenone prostaglandins that generally act as resolution lipids [7]. Thus, PGE_2_ induction by RG might represent both neuroprotective and neurotoxic processes and this issue requires additional study. 

Activation of TLR signaling pathways induces the members of the major mitogen-activated protein kinase (MAPK) subfamilies, including p38 and Jun N-terminal kinase (JNK) [6]. MAPK activation was previously attributed only to pro-inflammatory phase of TLR signaling, but recently it has been shown that enhanced and prolonged activation of MAPKs was an important mechanism underlying IL-10 production by LPS-stimulated macrophages [40,41]. Our results showed that rosiglitazone induced p38 MAPK, but not JNK, phosphorylation, and the effect was enhanced in the presence of LPS or PIC in neurons, indicating possible molecular mechanism responsible for the observed alterations in IL-10 and PGE_2_ production. 

## 4. Materials and Methods 

### 4.1. Reagents

LPS (Sigma-Aldrich, St. Louis, MO, USA), rosiglitazone (Sigma-Aldrich), streptomycin–penicillin, trypsin, EDTA, fetal bovine serum and Culture medium Dulbecco’s Modified Eagle Medium (DMEM) were from PanEco (Moscow, Russia). Antibodies against COX-2 (Cell Signaling Technology, D5H5, Danvers, MA, USA), phospho-p38 (Cell Signaling Technology, 4511), p38 (Cell Signaling Technology, 9212), p-JNK (cat.no sc-12882), JNK (cat.no sc-571), and β-actin (cat.no sc-47778) (Santa Cruz Biotechnology, CA, USA (SCBT)); secondary horseradish peroxidase conjugated antibodies (anti-rabbit, anti-mouse, and anti-goat) (SCBT and CST); Western Blotting Substrate ECL (Thermo Fisher Scientific, MA, USA); and ELISA kits for PGE_2_, TNFα and IL-10 (Thermo Fisher Scientific) were also used.

### 4.2. Astrocytes Primary Cell Culture

Cells were obtained from 1 or 2 day old pups of Wistar rats. Primary cultures of rat astrocytes were obtained from neonatal Wistar rats according to a protocol used previously [24]. In brief, the animals were aseptically decapitated, and the brains were isolated, washed in ice-cold Puck’s buffer (137.0 mM NaCl, 5.4 mM KCl, 0.2 mM KH_2_PO_4_, 0.17 mM Na_2_HPO_4_, 5.0 mM glucose, 58.4 mM sucrose, pH 7.4) and minced against meshes of 250 and 136 μm size, then the tissue fragments were placed into culture flasks. The material was supplied with DMEM (1 g/L d-glucose, 10% bovine fetal serum (FBS), 50 units/mL streptomycin, 50 μg/mL penicillin) and incubated at 37 °C, 5% CO_2_, 10% humidity. Five days later, cultures were shaken to detach microglial cells and given fresh portions of media with the same composition. The cells were cultured for an additional 6 days with media being changed every 2 days. After monolayer formation, the cells were treated with trypsin, then the cells were plated into 6-well culture plates at 750,000 cells/well. The cells were used for experiments after two days.

### 4.3. Primary Cultures of Rat Neuron Cortical Cells

To obtain the cultures cerebral hemispheres were isolated from 18-day-old Wistar rat embryos and washed in Hanks’ balanced salt solution without Ca^2+^ and Mg^2+^ (PanEco), cleared of blood vessels, dissected in trypsin EDTA solution (PanEco), then incubated in trypsin EDTA solution for 20 min at 37 °C. After that trypsin was inactivated with 10% fetal bovine serum (FBS) (PanEco) in Hanks’ solution. The preparation was washed twice with Hanks’ solution and suspended in Minimum Essential Medium (MEM) (PanEco) with 10% FBS and 100 U/mL penicillin–streptomycin (PanEco). The resulting suspension was centrifuged for 2 min at 400 g. The cells were then resuspended in MEM containing the additives listed above and dispensed into 6- (SPL Life Sciences, ROK, Naechon-Myeon, Pocheon-si, Korea) and 96-well (Nunc, Thermo Fisher Scientific, Waltham, MA USA) plates pretreated with poly-l-ornithine (Sigma, St. Louis, MO, USA) with the density of 1.2 × 10^5^ cells/cm^2^. The plates were pretreated with a 0.1 mg/mL poly-l-ornithine solution for several hours, then washed once with sterile water. Cultures were maintained in an incubator (SHEL LAB, Cornelius, OR, USA) at 37 °C, 90% humidity, 5% CO_2_ for 24 h, then the medium was replaced with Neurobasal Medium (NBM) (Gibco, Thermo Fisher Scientific, 168 Third Avenue, Waltham, MA, USA) with 2% B-27 Serum Free Supplement (Gibco), 100 U/mL penicillin-streptomycin and 1% GlutaMAX (Gibco). Afterwards, cultures were maintained in the incubator for 10–12 days. Half of the medium volume was refreshed every 3 days.

### 4.4. Western Blotting

Western blotting was used to evaluate the changes in the phosphorylation of the MAP kinases studied. The procedure was carried out in the same manner as described in our earlier work [42]. Cultured cells were lysed in RIPA buffer (Sigma) containing cocktails of protease and phosphatase inhibitors (Sigma). Then, the protein concentration was measured using DC Protein Assay Kit (Bio-Rad, Hercules, CA, USA). Primary antibodies to the following proteins were used: p-p38 (cat.no #4511), and p38 (cat.no #9212) (Cell Signaling Technology, (CST)); p-JNK (cat.no sc-12882), JNK (cat.no sc-571), and β-actin (cat.no sc-47778) (Santa Cruz Biotechnology, (SCBT)); and secondary horseradish peroxidase conjugated antibodies (anti-rabbit, anti-mouse, and anti-goat) (SCBT, CST). Membranes were developed using SuperSignal West Femto Maximum Sensitivity Substrate or SuperSignal West Pico Chemiluminescent Substrate (Thermo Scientific, MA, USA). Luminescence was detected by means of ChemiDoc XRS+ system (Bio-Rad), and the luminescence intensity was calculated with Image Lab 3.0 software (Bio-Rad).

### 4.5. Determination of TNFα, IL-10 and PGE_2_ by Enzyme-Linked Immunoassay

After the experiments, the supernatant was collected and stored at −70 °C for the further analysis. The levels of released PGE_2_, TNFα and IL-10 were determined using an enzyme-linked immunoassay commercial kits and Synergy H4 plate reader (BioTek, Winooski, VT, USA) following the manufacturer’s instructions.

### 4.6. Experimental Data Analysis and Statistics

Data are expressed as mean ± SEM. All experiments were reproduced three times. The data are presented as mean ± standard error obtained from three independent experiments. Data were subjected to an ANOVA test. The difference was considered statistically significant at *p* < 0.05.

## 5. Conclusions

Taken together, our data show that RG acts on astrocytes as an anti-inflammatory and a pro-resolution substance by reducing TNFα and inducing IL-10 release, respectively, and as a pro-resolution substance in neurons. To our knowledge, this is the first report demonstrating that rosiglitazone enhances IL-10 release in TLR-stimulated cultured neurons. Our data indicate that RG influences p38 MAPK phosphorylation that corresponds to the mechanisms of p38MAPK/IL-10 regulatory pathway known for macrophages. However, we cannot exclude the possibility that RG might influence mRNA stability, in a fashion similar to that in astrocytes [21]. This issue needs further investigations. It is important to add that RG did not change the time-course of TLR-mediated IL-10 release in neurons but enhanced its production. The same effect was observed for modulation of PGE_2_ synthesis. Although the detailed mechanisms of these findings are still to be investigated, our data allow supposing that rosiglitazone may be a promising substance for neuroinflammation therapy as an inducer of resolution in neurons and astrocytes. 

## Abbreviations

TLR	toll-like receptor
PIC	poly I:C
LPS	lipopolysaccharide
TNFα	tumor necrosis factor alpha
MAPK	mitogen-activated kinase
RG	rosiglitazone
IL-10	interleukin-10
